# Modulation of the Left Prefrontal Cortex with High Frequency Repetitive Transcranial Magnetic Stimulation Facilitates Gait in Multiple Sclerosis

**DOI:** 10.1155/2015/251829

**Published:** 2015-09-02

**Authors:** Amer M. Burhan, Priya Subramanian, Luljeta Pallaveshi, Brittany Barnes, Manuel Montero-Odasso

**Affiliations:** ^1^Division of Geriatric Psychiatry, Schulich School of Medicine and Dentistry, University of Western Ontario, London, ON, Canada N6A 3K7; ^2^Department of Psychiatry, University of Western Ontario, London, ON, Canada N6A 3K7; ^3^“Gait and Brain Lab”, Parkwood Hospital and Lawson Health Research Institute, London, ON, Canada N6C 0A7; ^4^“Gait and Brain Lab”, Lawson Health Research Institute, and Department of Medicine, Division of Geriatric Medicine, and Department of Epidemiology & Biostatistics, Schulich School of Medicine and Dentistry, University of Western Ontario, London, ON, Canada N6A 3K7

## Abstract

Multiple Sclerosis (MS) is a chronic central nervous system (CNS) demyelinating disease. Gait abnormalities are common and disabling in patients with MS with limited treatment options available. Emerging evidence suggests a role of prefrontal attention networks in modulating gait. High-frequency repetitive transcranial magnetic stimulation (rTMS) is known to enhance cortical excitability in stimulated cortex and its correlates. We investigated the effect of high-frequency left prefrontal rTMS on gait parameters in a 51-year-old Caucasian male with chronic relapsing/remitting MS with residual disabling attention and gait symptoms. Patient received 6 Hz, rTMS at 90% motor threshold using figure of eight coil centered on *F*_3_ location (using 10-20 electroencephalography (EEG) lead localization system). GAITRite gait analysis system was used to collect objective gait measures before and after one session and in another occasion three consecutive daily sessions of rTMS. Two-tailed within subject repeated measure *t*-test showed significant enhancement in ambulation time, gait velocity, and cadence after three consecutive daily sessions of rTMS. Modulating left prefrontal cortex excitability using rTMS resulted in significant change in gait parameters after three sessions. To our knowledge, this is the first report that demonstrates the effect of rTMS applied to the prefrontal cortex on gait in MS patients.

## 1. Introduction

Multiple Sclerosis (MS) is a central nervous system (CNS) demyelinating disease mainly affecting young adults. In 2008, the WHO estimated that 2–2.5 million people around the world have MS [1]. Individuals with MS experience different symptoms depending on parts of the CNS involved in disease exacerbations. The most common neurological deficits manifested include visual loss, extraocular movement impairment, paresthesias, executive dysfunction, and impairment in information processing speed, which leads to cognitive and gait abnormalities. Gait issues in MS are a significant source of disability [2]. Research reports that individuals with MS have significant impairment in gait velocity, cadence, and step length compared to healthy controls [3–5].

Transcranial Magnetic Stimulation (TMS) is a relatively noninvasive procedure whereby a magnetic field is generated by a powerful electric current passing through a hand held coil attached to a capacitor. Repetitive Transcranial Magnetic Stimulation (rTMS) is a promising therapeutic tool for various neurological and psychiatric diseases [6]. Although single and paired pulse TMS have been used as a diagnostic tool in MS, there are only a few studies demonstrating the effect of rTMS as a therapeutic tool in this patient population. Centonze et al. [7] investigated whether rTMS can modify spasticity by using high-frequency (5 Hz) and low-frequency (1 Hz) rTMS protocols in 19 patients with relapsing-remitting MS and lower limb spasticity. Koch et al. [8] demonstrated that rTMS of the motor cortex may be a useful approach to treat cerebellar impairment in individuals with MS. A recent study by Kumru et al. [9] demonstrated that 15 daily sessions of high-frequency rTMS can improve motor scores, walking speed, and spasticity in the lower limbs in incomplete spinal-cord injury patients. Although the effect of TMS on cognitive and motor functions in other neurological populations such as Parkinson's disease has been explored [10], we are not aware of any study that explored the effect of rTMS on gait in MS patients.

The current study aimed to explore the effect of rTMS for gait impairment in an MS patient.

## 2. Case Presentation

**Figure 1:**
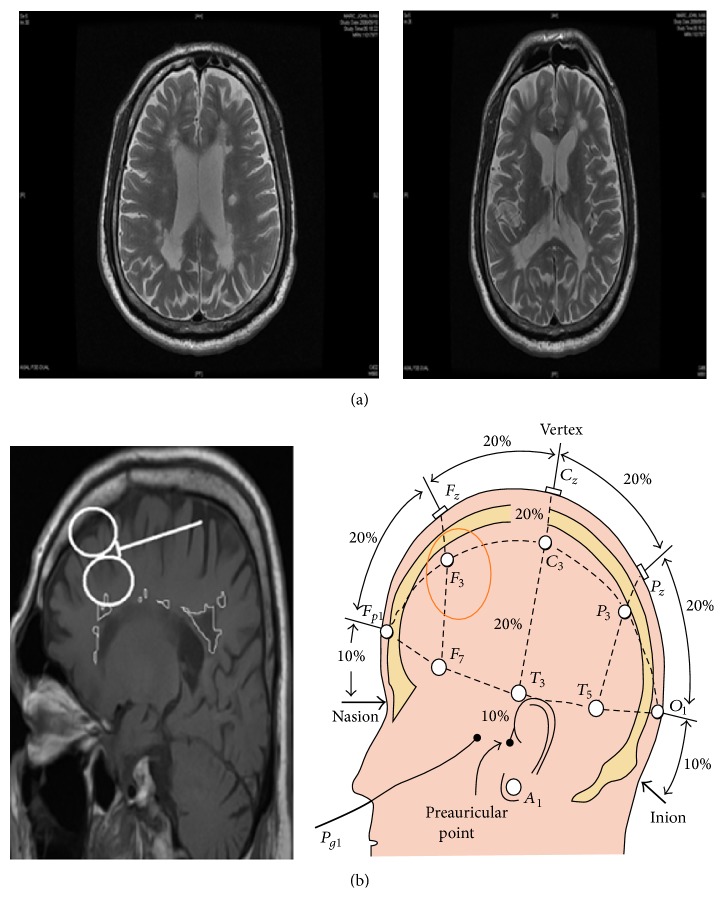
(a) Patient's *T*_2_ MRI transverse image showing several patches of white matter hyper-intensities representing demylinating lesions. (b) Patient's *T*_1_ MRI sagittal image (on the left) showing approximate TMS coil location using the 10-20 international EEG lead localization system (on the right). White matter lesions are marked for demonstration on the *T*_1_ sagittal image.

A 51-year-old Caucasian male with 4-year history of MS was referred to our neuropsychiatry memory clinic by his family doctor for a cognitive assessment and intervention. His course is best described as relapsing and remitting in nature. He had left upper and lower limb spastic weakness, double vision, jerky eye pursuit, and some cognitive deficits mainly in attention. Depression and anxiety screening was negative, although patient reported frustration with his “slowed thinking.” Patient's MRI showed significant white matter involvement including left prefrontal and parietal white matter, areas that are relevant to attention (Figure 1). On cognitive assessment using the Montreal Cognitive Assessment scale (MoCA) original English version [11] patient scored 23 out of 30 missing one point on letter fluency, 4 points on delayed recall though he was helped with cueing, one point on copying the cube, and one point on sentence repetition. His categorical fluency was 12 animals in one minute while letter fluency was 7 words starting with letter “F” in one minute.

Based on case reports of benefit of Cholinesterase inhibitors in MS patients [12] we offered a trial of Rivastigmine patch which was ineffective. Patient inquired about TMS as an enhancer of brain function. We speculated that enhancing left prefrontal cortical activation may enhance attention. We offered a trial of five daily sessions of high-frequency rTMS applied to left dorsolateral prefrontal location. Although patient reported subjective improvement in “thinking,” there was no significant effect of this intervention on cognitive function except for reduction in the time needed to complete Trail A from 122 to 60 seconds. Patient spontaneously reported walking faster and with less effort. This was in keeping with the finding of faster completion of Trail A, which is largely a speed of processing task. He requested a trial of rTMS for his walking. After we discussed potential benefits and potential known risks including head discomfort, headaches, and seizures [13], we obtained an informed consent from the patient as per our institution's standard policy.

We used a figure of eight air film-cooled coil attached to Magstim Super Rapid 2 TMS device (Magstim, UK). This coil produces a cone shape magnetic field with a 1 centimeter square peak that can penetrate around 2 cm into the cortex of the brain. Hundred percent of the machine output produces about 3 tesla magnetic field power. The magnetic field passes unimpeded to the cortex and induces a local current. It is thought that this current is frequency dependent and results in modulation of cortical interneurons. A higher frequency magnetic pulse (3 or more cycles per second) is usually excitatory (which enhances glutamatergic interneurons activity whereby it results in lower threshold of the cortical projection neurons) while low frequency magnetic pulse (below 3 cycles per second) is usually inhibitory in nature (which enhances GABAergic interneurons activity and results in higher threshold for firing of cortical projection neurons) [14, 15].

We used 10-20 international EEG lead localization system to identify *F*_3_ location which usually corresponds to left dorsal lateral prefrontal cortex (L-DLFC) [16]. Patient received high-frequency 6 Hz rTMS at 90% resting motor threshold, defined as the minimum amount of energy needed to induce a visible muscle twitch in the first dorsal interosseous muscle on more than 50% of the trials while the patient's hands are resting. Each session involved delivering 1200 pulses divided to several trains based on safety parameters built in to MAGSTIM Rapid2 machine (MAGSTIM, UK). In general, treatment was well tolerated except for some local scalp discomfort.

Patient received one session and three consecutive daily sessions of rTMS treatment separated by 3 days. We administered rTMS using the same parameters for both sessions. Gait data were collected at 2 points of time: (a) before (baseline 1) and immediately after one rTMS session and (b) before (baseline 2) and immediately after 3 consecutive daily rTMS sessions. The gait pattern was assessed during three consecutive trials at a usual gait.

Gait performance was assessed using an electronic walkway system (GAITRite System http://www.gaitrite.com/) that is 600 cm in length and 64 cm in width. As participants walk along the mat, imbedded sensors are activated by the pressure of their feet and deactivated when the pressure is released. A computer processed the footsteps, providing data for both spatial and temporal parameters. Start and end points were marked on the floor with tape 1 m from either end of the mat to avoid the recording of acceleration and deceleration phases. Participant performed 1 practice trial walking on the mat to familiarize himself with the protocol. Ambulation time (time elapsed between first contact of the first and the last footfalls, measured in seconds), gait velocity (cm/s), stride time (ms), cadence (number of full cycles taken by the pair of feet per minute), and stride time variability (percentage of coefficient of variation (%CoV)), the principal gait measures of interest, were measured over three trials which consisted of walking the length of the mat at a self-selected usual pace.

A descriptive data analysis (means and proportions) was performed on quantitative data using SPSS software package 21.0 (SPSS Inc., Chicago, IL). We used *t*-tests repeated measures within subject for multiple comparisons of pre- and post-rTMS sessions. Comparisons were made for outcomes at each time point with respect to the baseline to examine any significant differences. We set the level of statistical significance at *p* < 0.05. The *t*-test for paired data was used for statistical analysis.

Overall, the results indicated that patient had significant improvement in his gait parameters after being treated with rTMS.  Table 1 indicates the gait parameters measured before and after 1 rTMS session and before and after the 3 consecutive rTMS sessions.

There were no statistically significant differences before and after 1 rTMS session in ambulation time and velocity; however, cadence was significant (*t*(2) = −4.99, *p* < 0.05). Statistically significant differences were found before and after 3 consecutive daily rTMS sessions in ambulation time (*t*(2) = 8.32, *p* < 0.05), velocity (*t*(2) = −4.59, *p* < 0.05), and cadence (*t*(2) = −7.57, *p* < 0.05).

**Figure 2:**
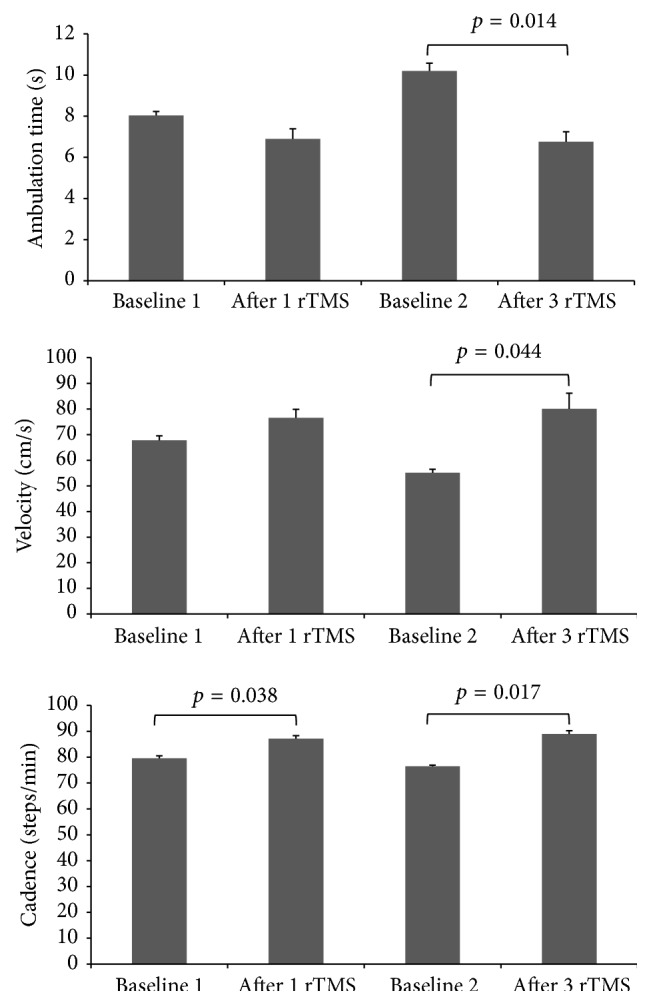
Mean data from three usual gait trials showing ambulation time, velocity, and cadence at baseline, after one rTMS session, at baseline 2 (three days after one rTMS session), and after three consecutive daily rTMS sessions. Ambulation time was significantly shorter and velocity was significantly faster only after 3 rTMS sessions while cadence was significantly higher after both 1 and 3 rTMS sessions (see Table 1 for details).

Analysis of data showed that stride time variability, measured as the time elapsed between the first contact of two consecutive footfalls of the same foot, was decreased after the 1 rTMS session from 5.02% CoV to 4.6% CoV. However, it was increased after patient received 3 consecutive rTMS sessions from 4.64% CoV to 5.34% CoV; see Figure 2 for graphic representation of the GAITRite data.

## 3. Discussion

In this study we report shorter ambulation time and faster velocity, in response to three rTMS daily sessions in addition to increased cadence after one and three rTMS sessions in a patient with 4-year history of relapsing and remitting MS presenting with cognitive and gait abnormalities. The above gait parameters are commonly affected in MS patients and cause significant functional impairment and risk for falls. To our knowledge, this is the first report that demonstrates the effect of rTMS applied to the prefrontal cortex on gait in MS patients. The mechanism underlying rTMS effect on gait is not fully understood but it is likely related to enhancing excitability of the left prefrontal cortex which in turn exerts control over volition aspect of gait. The prefrontal cortex is functionally connected to the caudate. There is evidence of increased Dopaminergic transmission in the caudate as a result of prefrontal cortical stimulation with TMS [17], which might be one possible mechanism of this effect.

The magnitude of changes observed on gait velocity, in the range of 10 cm/sec, is clinically meaningful and is similar to the gait improvements seen after exercise intervention protocols [18, 19]. In addition, these velocity improvements are unlikely to be related to learning effects since we have demonstrated the test-retest reliability of quantitative gait assessments after repeated measurement and no changes related to learning effects were described [20]. The ability to modify gait abnormalities in MS using cortical stimulation is an exciting prospect but requires further study to identify which aspects of gait are modifiable and the implication of that on function and fall risk.

Previous research has demonstrated that magnitude and duration of the rTMS effects seem to depend upon the total number of stimuli, with longer periods of rTMS inducing a higher consistency in cortical excitability [14]. In this case study we found an increase in the ambulation time, velocity, and cadence in somewhat of a dose dependent fashion. On the other hand, although stride time variability was decreased after one rTMS session, it did increase after three rTMS sessions. This has implication on fall risk because there is a positive correlation between stride time variability and fall risk [21, 22]. This might be related to ambulation time and velocity: that is, the faster the gait the more stride time variability in this case and hence more risk of falls.

There are several limitations in this study. As a single case study its findings cannot be generalized due to variability in clinical presentation and lesion location in MS patients. Also, we used a probabilistic localization system to place the TMS coil on the prefrontal cortex, namely, the 10-20 international EEG lead localization system; hence, we cannot be sure about the precise anatomical area being stimulated beyond approximation. This case study helps identify the impact of one rTMS session and the effect of three rTMS daily sessions on gait. There was a short time difference (3 days) between the one rTMS session and the beginning of the three consecutive daily rTMS sessions. This resulted in a new baseline being established, which was lower in ambulation time and velocity compared to the first baseline. The occurrence of this difference is hard to interpret; hence, further investigation is needed. Furthermore, we could not assess the long term impact of rTMS on gait because we did not provide any follow-up for this case. In this case study, we chose to stimulate the dominant left side assuming that it is more related to volition. The effect of nondominant prefrontal cortex rTMS was not explored.

The results of this case study indicate that modulating left prefrontal cortex excitability using high-frequency rTMS resulted in significant change in gait parameters in an MS patient with minimal discomfort. This indicates potential utility of this noninvasive brain stimulation technique in modulating gait parameters in this patient population. Further research is needed to clarify the role of rTMS as a therapeutic tool in MS patients using appropriate clinical trial design to address questions raised in this case report.

## Figures and Tables

**Table 1:** Gait parameters scores before and after 1 rTMS session and 3 consecutive daily rTMS sessions.

Gait parameters	One rTMS Session					Three consecutive rTMS sessions				
	Mean changes			SE^∧^	*p* value	Mean changes			SE^∧^	*p* value
	Before	After	Mean difference			Before	After	Mean difference		
Ambulation time (sec)	8.04	6.90	−1.14	0.68	0.237	10.19	6.76	−3.62	0.41	0.014^*∗*^
Velocity (cm/sec)	67.80	76.53	8.73	2.86	0.093	55.10	80.07	25.57	5.43	0.044^*∗*^
Cadence (steps/min)	79.60	87.13	7.53	1.51	0.038^*∗*^	76.47	88.97	12.50	1.65	0.017^*∗*^
Step length left (cm)	60.88	61.19	.312	1.92	0.886	51.49	63.12	−11.62	2.85	0.055
Step length right (cm)	42.45	45.14	2.68	2.36	0.373	35.37	44.79	9.43	5.03	0.201
Step time left (sec)	0.74	0.68	−0.06	0.02	0.109	0.78	0.68	−0.10	0.01	0.030^*∗*^
Step time right (sec)	0.76	0.70	−0.06	0.01	0.010^*∗∗*^	0.79	0.66	−0.12	0.02	0.032^*∗*^
Cycle time left (sec)	1.52	1.38	−0.14	0.03	0.031^*∗*^	1.57	1.34	−0.23	0.02	0.011^*∗*^
Cycle time right (sec)	1.52	1.37	−0.14	0.03	0.036^*∗*^	1.57	1.36	−0.21	0.03	0.027^*∗*^
Stride length left (cm)	103.2	106.6	3.39	3.14	0.393	96.93	108.39	21.46	6.52	0.081
Stride length right (cm)	104.7	106.5	1.83	1.56	0.363	87.16	108.74	21.58	7.75	0.109
Swing time left (sec)	0.50	0.47	−0.03	0.01	0.058	0.51	0.48	−0.03	0.01	0.135
Swing time right (sec)	0.58	0.49	−0.08	0.01	0.003^*∗∗*^	0.53	0.46	−0.06	0.02	0.087
Stance time left (sec)	1.02	0.91	−0.11	0.02	0.030^*∗*^	1.06	0.86	−0.20	0.01	0.003^*∗∗*^
Stance time right (sec)	0.94	0.89	−0.05	0.03	0.174	1.04	0.89	−0.15	0.03	0.035^*∗*^

SE^∧^: standard error; ^*∗*^ *p* < 0.05; ^*∗∗*^ *p* < 0.01.
